# Disrupted seasonal biology impacts health, food security and ecosystems

**DOI:** 10.1098/rspb.2015.1453

**Published:** 2015-10-22

**Authors:** T. J. Stevenson, M. E. Visser, W. Arnold, P. Barrett, S. Biello, A. Dawson, D. L. Denlinger, D. Dominoni, F. J. Ebling, S. Elton, N. Evans, H. M. Ferguson, R. G. Foster, M. Hau, D. T. Haydon, D. G. Hazlerigg, P. Heideman, J. G. C. Hopcraft, N. N. Jonsson, N. Kronfeld-Schor, V. Kumar, G. A. Lincoln, R. MacLeod, S. A. M. Martin, M. Martinez-Bakker, R. J. Nelson, T. Reed, J. E. Robinson, D. Rock, W. J. Schwartz, I. Steffan-Dewenter, E. Tauber, S. J. Thackeray, C. Umstatter, T. Yoshimura, B. Helm

**Affiliations:** 1 Institute for Biological and Environmental Sciences, University of Aberdeen, Aberdeen, UK; 2 Department of Animal Ecology, Nederlands Instituut voor Ecologie, Wageningen, The Netherlands; 3 Research Institute of Wildlife Ecology, University of Vienna, Vienna, Austria; 4 Rowett Institute of Nutrition and Health, University of Aberdeen, Aberdeen, UK; 5 School of Psychology, University of Glasgow, Glasgow, UK; 6 Centre for Ecology and Hydrology, Penicuik, Midlothian, UK; 7 Department of Entomology, Ohio State University, Columbus, OH, USA; 8 Centre for Ecology & Hydrology, Lancaster Environment Centre, Library Avenue, Bailrigg, Lancaster, UK; 9 School of Life Sciences, University of Nottingham, Nottingham, UK; 10 Department of Anthropology, Durham University, Durham, UK; 11 Nuffield Department of Clinical Neurosciences, University of Oxford, Oxford, UK; 12 Max Planck Institute for Ornithology, Seewiesen, Germany; 13 Department of Arctic and Marine Biology, University of Tromso, Tromso, Norway; 14 Department of Biology, The College of William and Mary, Williamsburg, VA, USA; 15 Department of Zoology, Tel Aviv University, Tel Aviv, Israel; 16 Department of Zoology, University of Delhi, Delhi, India; 17 School of Biomedical Sciences, University of Edinburgh, Edinburgh, UK; 18 Department of Ecology and Evolution, University of Michigan, Ann Arbor, MI, USA; 19 Department of Psychology, Ohio State University, Columbus, OH, USA; 20 Aquaculture and Fisheries Development Centre, University of College Cork, Cork, Ireland; 21 School of Psychiatry and Clinical Neurosciences, University of Western Australia, Perth, Australia; 22 Department of Neurology, University of Massachusetts Medical School, Worcester, MA, USA; 23 Department of Animal Ecology and Tropical Biology, University of Wuerzburg, Wuerzburg, Germany; 24 Department of Genetics, University of Leicester, Leicester, UK; 25 Agroscope, Tanikon, CH-8356 Ettenhausen, Switzerland; 26 Department of Applied Molecular Biosciences, University of Nagoya, Nagoya, Japan

**Keywords:** annual, fitness, desynchrony, one-health, biological rhythm, circannual

## Abstract

The rhythm of life on earth is shaped by seasonal changes in the environment. Plants and animals show profound annual cycles in physiology, health, morphology, behaviour and demography in response to environmental cues. Seasonal biology impacts ecosystems and agriculture, with consequences for humans and biodiversity. Human populations show robust annual rhythms in health and well-being, and the birth month can have lasting effects that persist throughout life. This review emphasizes the need for a better understanding of seasonal biology against the backdrop of its rapidly progressing disruption through climate change, human lifestyles and other anthropogenic impact. Climate change is modifying annual rhythms to which numerous organisms have adapted, with potential consequences for industries relating to health, ecosystems and food security. Disconcertingly, human lifestyles under artificial conditions of eternal summer provide the most extreme example for disconnect from natural seasons, making humans vulnerable to increased morbidity and mortality. In this review, we introduce scenarios of seasonal disruption, highlight key aspects of seasonal biology and summarize from biomedical, anthropological, veterinary, agricultural and environmental perspectives the recent evidence for seasonal desynchronization between environmental factors and internal rhythms. Because annual rhythms are pervasive across biological systems, they provide a common framework for trans-disciplinary research.

## 1. Introduction

Biological rhythms are ubiquitous in nature and occur on several temporal scales. Daily rhythms are important for the coordination of physiological, immunological and behavioural processes within organisms as well as for biotic interactions. The proper functioning of these rhythms is disrupted by modern human lifestyles—including sleep deprivation, light at night, jet lag and shift work [1,2], which induce temporal mismatches between the environment and circadian biology, and have detrimental effects on health and well-being. Problems of mismatch extend beyond daily rhythmicity. New research by Dopico *et al.* [3] has elegantly demonstrated massive seasonal changes in human immunity and physiology, adding to evidence for marked annual rhythms in the vast majority of organisms [4,5]. The disruption of annual rhythms under global climate change has potentially dramatic consequences for the health of animals, humans and ecosystems. Whether or not organisms can adapt to changing seasonality depends on the regulation of their annual rhythms. Principally, annual rhythms could be: (i) *genetically programmed*, i.e. genotype-dependent responses to the environment resulting from evolutionary adaptation to predictable annual change; (ii) *direct environmental* effects, e.g. accelerated growth owing to longer light hours in the day; or (iii) *coincidental*, e.g. human rhythms arising as a consequence from holidays.

This review will focus on genetically programmed, internal cell- and tissue-based mechanisms, which are predicted to track changes in environmental seasonality. In many species, tissue function is reprogrammed between subjective winter and summer states, generating endogenous rhythms that approximate a year (i.e. circannual rhythms) [3,6–9]. The existence of innate circannual rhythmicity has been demonstrated when organisms, from unicells to vertebrates, are maintained in constant environmental conditions for many years [6–11]. Species with genetically programmed annual rhythmicity occur globally, from high latitudes to the equator, and even in apparently ‘constant’ environments such as the deep sea [6]. Genetic programming is seen to be adaptive because it is *pre-emptive* and serves to predict and prepare organisms for alternations in seasonal environmental conditions [12–14].

In humans, the evidence in support of seasonal effects on disease risk, physiology and immune function is pervasive ([3,15–17]; electronic supplementary material, tables S1 and S2) and suggests present-day implications of evolutionarily inherited and refined mechanisms [5]. During the twentieth century, our species has developed technologies that allow precise photic and climate control over our living environments, and humans in developed societies now spend the vast majority of their lives in conditions that mimic ‘summer-like’ environments [15]. These so-called *eternal summers* are characterized by light and temperature conditions that lack seasonal rhythmicity. Presently, many of us no longer live in accordance with the naturally occurring variation in geophysical rhythms. The consequences of such modified environmental seasonality on human health are still being elucidated.

Additionally, human activities are also affecting seasonality in a wider ecological context, with implications for disease risk, ecosystem health and food security. In agricultural and natural ecosystems, there is growing evidence that seasonal patterns and ecological interactions are disrupted by global climate change [18]. Disruptions in annual rhythms are expected to become progressively more prevalent and detrimental consequences have already been documented [19,20]. Nevertheless, there is a surprising lack of data to determine if, and how seasonally generated and regulated functions can adapt [21,22] to altered environmental conditions. Thus, an overarching, integrated scientific understanding is needed of the mechanisms that underlie seasonality, including cyclical biology of humans and of the consequences for all organisms as they adapt to disrupted seasonality and a fast changing climate. Addressing the arising practical challenges requires an integrated, cross-disciplinary approach, as exemplified by the ‘one health’ initiative for advancing healthcare for humans, animals and the environment [23,24].

Here, we review current examples of disruption between seasonal environmental conditions and internal timing mechanisms. From this basis, we further emphasize the pervasiveness of seasonality (with a focus on humans). Finally, we outline potential threats of disruption for humans, industry, and managed and natural ecosystems, with a call for the development of a synergistic research agenda for seasonal biology.

## 2. Scenarios of seasonal disruption

**Figure 1.**
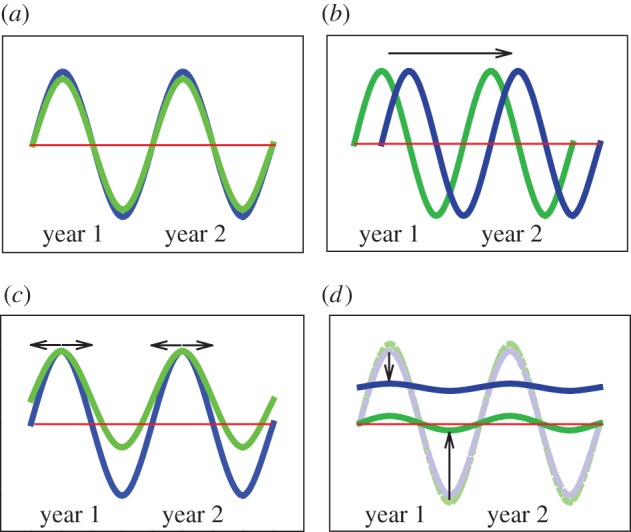
Schematic of annual rhythms. (*a*) All organisms on earth have evolved to time their physiology and behaviour (internal rhythm; blue) with seasonal changes in local climates/resources (external rhythm; green line). These internal rhythms can precede or follow those of resources, but for adaptive timing, the internal and external rhythms need to match. There are three theoretical scenarios that can account for disruptions of the match between seasonal timing to local climates: (*b*) phase shifts between internal and external rhythms; (*c*) increased (or decreased) duration of favourable environmental conditions (e.g. rising above the red line, which could indicate a rise in minimum temperature and day length); or (*d*) reduction of the amplitude (e.g. under ‘eternal summers’) and/or change in mean levels of seasonal rhythms. Red lines indicate the average seasonal mean of the rhythms in panel (*a*) for reference. Arrows indicate changes in rhythms. (Online version in colour.)

Seasonal changes in environmental variables play a significant role in the regulation of many physiological and behavioural processes. Annual changes in day length, temperature and rainfall can all act as cues; they provide vital information used in the timing of seasonal behaviour and the synchronization of internal rhythms (electronic supplementary material, figure S1 and figure 1). In this way, organisms' behaviour and physiology is timed to optimize fitness in a given season [12,13,19]. However, the use of these cues to synchronize (entrain) biological rhythms is effective only to the extent that temporal relationships between the external and internal rhythms are predictable. Timing systems are therefore highly vulnerable to changes in the constellation of environmental factors under which they have evolved (figure 1). Changes in the seasonal timing of organismal functions provided some of the earliest evidence for biological effects of global climate change [25]. Such changes have now been documented across a wide range of habitat types and taxonomic groups (electronic supplementary material, figure S2 and [26]). Crucially, species and populations of wild plants and animals have demonstrated different rates of change in their overt seasonality [26].

There are multiple, mutually non-exclusive and potentially interactive mechanisms by which altered external cues could disrupt the relationship between external and internal rhythmicity. For example, climate-change-induced shifts in the timing of the seasons can result in an abnormally delayed or advanced internal rhythm with respect to the environment (figure 1*b*). Such mismatches in the timing of critical life-history events can have a large impact on the reproductive output of plants and animals. Alternatively, disruptions might be induced if the durations of specific phases of an external rhythm become extended (e.g. longer growing season) or reduced (e.g. shorter time of snow cover). This could occur, for example, if local minimal winter temperatures increase, resulting in higher means in local annual temperatures with reduced amplitude of seasonal differences (figure 1*c*). A scenario with changes in the amplitude of seasonal differences is particularly relevant for humans (figure 1*d*). Cultural developments, including the ability to control local environmental conditions through the use of fire at night for heat and light, marked the beginning of human manipulation of natural day–night cycles. The seasonality experienced by humans in developed societies (and that of some closely associated species) is already largely damped by modern artificially induced photic and indoor climate conditions reminiscent of eternal summers [15].

## 3. Molecular, cellular and physiological basis of seasonal time-keeping

A better understanding of how species respond to seasonal changes in their environment and how they can adapt to their disrupted seasonality requires knowledge of the mechanisms of seasonal time-keeping. Endogenous annual rhythmicity is evident in a wide range of species (e.g. protists [6], insects [27], plants [28], fishes [29], birds [30] and mammals [4]), but is especially strong and widespread among vertebrates, making it highly likely that seasonal genes and/or intracellular pathways are also involved in long-term changes in human physiology and behaviour. Broadly, in multicellular organisms, the mechanisms for regulating annual rhythms involve cellular and molecular timers that interact closely with refined input pathways for transmitting day length (photoperiod) and other environmental cues (electronic supplementary material, figure S3). Highly photoperiodic species have been extremely valuable for the identification of key genetic, cellular and neuronal circuits that regulate annual rhythms [31]. Direct evidence for endogenous seasonal rhythms in humans is scarce owing to the challenges of collecting data over multiple annual oscillations and the near impossibility of isolating subjects from exogenous influences for extensive time periods. Historical and contemporary studies have demonstrated that humans exhibit seasonal reproduction, hypothesized to be driven by internal mechanisms [32,33]. Over the past few centuries, there has been a decrease in seasonal patterns in humans [32,33] that could have resulted from a reduction in the seasonal amplitude or desynchronization from environmental cues. Nevertheless, these data suggest that exogenous cues (i.e. photoperiod) can entrain seasonal human responses [15,33,34]. In most mammals, annual changes in day length affect seasonal rhythmicity via the suprachiasmatic nucleus in the hypothalamus, and consequently, altering the nocturnal secretion of the hormone melatonin from the pineal gland ([35]; electronic supplementary material, figure S3*a*). Melatonin receptors are localized in many of the brain regions implicated in cognitive, affective and homeostatic processes, and thus seasonal changes in melatonin secretion regulate key genes required for the neuroendocrine control of physiology, immune function and behaviour [36–40]. Although exact photoperiodic input pathways vary among vertebrate groups, similar day length-induced changes in neuroendocrine brain regions (electronic supplementary material, figure S3*b*) occur in fishes [41] and birds [42], which would suggest that seasonal changes in day length act to regulate a common, evolutionarily ancient internal timing system [43]. Recent work suggests that the fundamental nature of this timer may depend on cyclical histogenesis [44] and/or epigenetic mechanisms [45,46], potentially operating in multiple tissues to form a circannual clock-network.

In this framework, the hypothalamus of the brain acts as an important interface for many processes that are relevant for health and physiology, coordinating seasonal changes in autonomic and endocrine function. Of note, seasonal timing in immune function is vital for the survival of many small seasonal animals such as rodents and birds [39–41]. Seasonal- or light-induced changes in a range of innate humoral and cell-mediated systems are present in a wide range of species (electronic supplementary material, table S2). A general finding is that short days which mimic a winter environment enhance some immune functions [39]; for species where winter is a time of increased pathological risk, immune function is increased presumably in anticipation of increased need. It is important to note that different aspects of immunity may be differently regulated across the seasons. For example, rodent and bird models have shown that short days enhance many aspects of cell-mediated immunity while suppressing other specific immune defences ([39,41,47]; electronic supplementary material, table S2). The precise molecular and cellular mechanisms involved in the seasonal restructuring of immune function are not well described, but recent evidence suggests that a common molecular switch occurs in hypothalamic regions and immune tissues (e.g. leucocytes; [3,48]). Recent developments in next-generation sequencing platforms, transcriptomics and proteomic analyses, provide a powerful means to identify the precise molecular and cellular pathways that underlie annual rhythms [3,44,46]. Once the mechanisms that govern such rhythms are identified, experiments can be devised for field-based or clinical settings to examine the relationship between external environment and internal timing.

## 4. Seasonality and human health

In many tropical and equatorial areas, including the savannah–woodlands where humans are thought to have originated, seasonality is highly pronounced although there is little variation in day length. Indeed, striking seasonal patterns in behaviour are observed in the migration and breeding rhythms of many equatorial species, such as the flowering of trees [49] and the reproductive seasonality of some primates and traditional human societies [50]. Although at a *population* level many species appear to breed asynchronously with respect to the calendar year, *individuals* tend to be seasonally cyclical [51,52] when studied over a full life history. Environmental stimuli that provide seasonal cues in the tropics are often related to local non-photic cues, such as rainfall patterns that in turn determine food abundance (electronic supplementary material, figure S1*c*). In the Pleistocene, humans radiated out of the tropics to higher latitudes where they encountered novel seasonal environments, in particular marked annual rhythms in day length and ambient temperature (electronic supplementary material, figure S1*a*–*c*). However, because the underlying internal mechanisms that govern annual rhythms preceded the hominin lineage, humans probably possess much of the ancient molecular and cellular machinery characteristics of other seasonal species, potentially including genetic variation in seasonality [53]. Even today, human reproduction is not evenly spread across the year ([33,54]; electronic supplementary material, figure S4*a*–*c*). For example, a recent analysis indicated that human birth pulses across the USA fluctuate seasonally with an amplitude of about 10% ([33]; electronic supplementary material, figure S4). Despite the decline in birth seasonality (figure 1*d*; cf. [15,17,32,33]), it is still substantial for a species that effectively lives under conditions of eternal summer in its immediate habitat. Notably, the amplitude of these rhythms and the timing of their maxima vary with latitude, which strongly suggests an underlying physiological regulatory mechanism. These patterns of birth pulses have longer-term relevance because of the strong evidence [55] that birth month has lifelong impacts on health, including the likelihood of general, psychiatric or neurological illnesses ([17,54]; electronic supplementary material, table S1). That these time-of-birth effects may reflect day-length-dependent mechanisms is signified by the observation that some diagnoses, e.g. schizophrenia and multiple sclerosis, show an inverse pattern in the Northern and Southern Hemispheres (electronic supplementary material, table S1).

**Figure 2.**
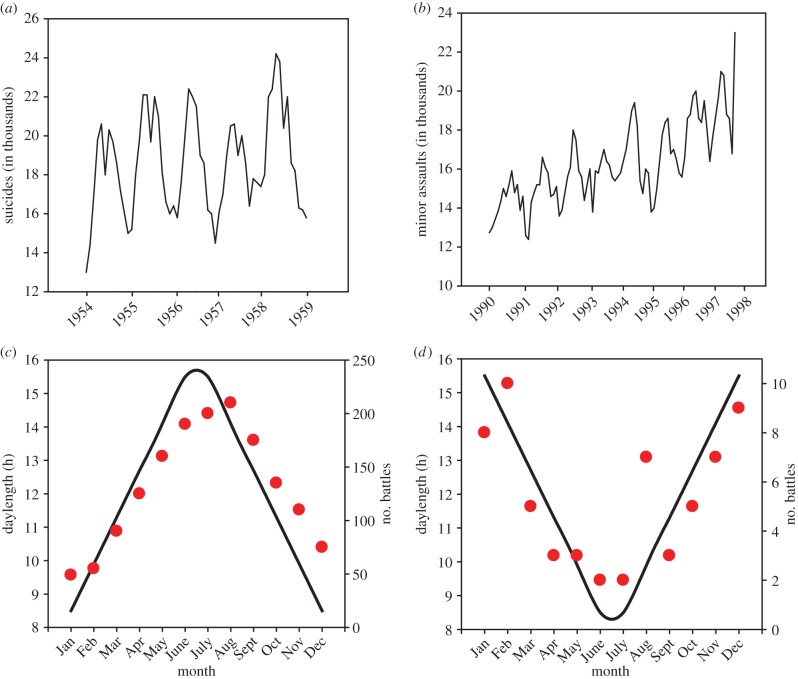
Seasonal patterns in humans. (*a*) Number of suicides in Japan was significantly greater in the spring compared with autumn seasons. (*b*) The number of minor assaults in England and Wales significantly increased during the summer compared with winter seasons. (*c*) Number of battles in the Northern hemisphere peaked in August and were at a minimum in January; (*d*) the inverse pattern occurs in the Southern Hemisphere with peaks of battles occurring December–February; troughs in July. Data from (*a,b*) were kindly provided by Daniel Rock; (*c,d*) adapted from [59]. (Online version in colour.)

Seasonal human morbidity is observed in non-infectious diseases, including heart disease [56], cerebrovascular disease [57] and lung cancer [58]. Behaviour-driven mortality is also seasonal; cycles in the monthly numbers of suicides are one of the oldest and most replicated findings, with most, but not all, studies finding a peak in late spring/early summer (figure 2*a*). Interestingly, aggression and other violent acts such as homicide have a marked seasonal pattern of occurrence nearly coincident with that found for suicide (figure 2*b*). Seasonal patterns in aggression are not limited to the individual level. Historical records indicate a strong rhythm in population-level forms of aggression measured in onset of battles ([59]; figure 2*c,d*). Whether rhythms represent simple direct responses to seasonal environmental changes and/or the manifestation of endogenous seasonal timing mechanisms, these findings indicate that seasonality continues to be an important factor in human lives.

Humans also show seasonal changes in immunity and in the occurrence of infectious diseases [47]. Changes in immunity include seasonal variation in cytokine production [60], bacterial killing activity [61] and response to vaccination [62]. The most comprehensive characterization so far of annual cycles in human immunity comes from a recent study by Dopico *et al.* [3]. This study presented extensive data from geographically and ethnically diverse human populations, including mRNA expression in white blood cells and adipose tissue, inflammatory markers and blood count data. It reports seasonal differences in the expression of up to 23% of genes in peripheral blood mononuclear cells. Furthermore, the seasonal patterns of gene expression were inverse in Australia relative to the USA and the UK. The reverse pattern in Australia supports the idea that in humans, as well as in other animals, some immune responses may be modulated by day length [63]. Seasonal infections thus probably mirror internal rhythms of immunity as well as patterns of exposure. The relative importance of these mechanisms as drivers of any human infection remains to be established.

## 5. Disrupted seasonality and infectious disease dynamics

One important area for future research is the effect of disrupted seasonality on the dynamics of infectious disease. A recent review has highlighted the unprecedented rate at which vector-borne diseases have changed over the past decade, and has alerted a wide audience to the impact of changes in the climate [64]. The review details consequences of modified environmental seasonality, for example release from severe winters at higher latitudes and extended phases of seasonal activity. Environmental seasonal drivers of disease incidence include climate-sensitive pathogen dissemination and survival; seasonal variation in host recruitment, contact rates and susceptibility, and seasonal changes in vector abundance [65,66]. Directly transmitted and epidemic-prone diseases such as influenza, measles, polio, rotavirus and cholera exhibit pronounced seasonality and substantial heterogeneity in time and space [65,66]. The dominant seasonal drivers vary not only by disease, but also by geography. The best understood examples of climate-driven infections are influenza and cholera. Influenza is best transmitted when temperature and humidity are low, yielding winter epidemics in temperate regions [67], whereas cholera outbreaks are intensified by local increases in temperature during El Niño events [68].

Vector-borne pathogens—including those responsible for malaria, dengue, Lyme disease, Chagas, West Nile and sleeping sickness—present some of the most notable examples of seasonally driven infection [65,66]. Vector-borne diseases are particularly sensitive to phenological change, because numerous aspects of vector behaviour, demography and population dynamics are crucially dependent on environmental conditions [69]. For example, the growth rate of malaria vector populations in Africa explodes during the rainy seasons owing to the expansion of vector-larval habitat [70,71]. Temperature also has a strong impact on larval development rate, survival and the duration of the gonotrophic cycle in a wide range of Diptera [72–75]. Further complexity is introduced by seasonality in host populations. Examples include seasonal variation in the immune responsiveness and nutritional quality of plant hosts to their vectors [72,75]; herd immunity in cattle to tick-borne *Babesia* [76] and demonstration that the timing of peak human exposure to West Nile virus in North America is driven by seasonal patterns of avian migration [77]. The complexity of the seasonal interactions between vectors, pathogens and hosts is probably responsible for the lack of consistent evidence on how climate change will influence vector-borne disease [64,71,74]. While there is compelling evidence that some vector-borne diseases are being enhanced by climate change (e.g*.* Lyme disease; [78,79]; avian malaria; [80]), there are several other examples of diseases that have failed to expand as originally predicted (e.g. malaria; [69]). The least understood seasonal drivers of infection are those potentially governed by rhythms of host susceptibility and susceptible recruitment [47], although recent data suggest that annual cycles in human immune pathways may indeed affect susceptibility to specific diseases [3]. In summary, there is a need for a detailed understanding of how climate will affect all aspects of the pathogen life cycle before the consequences for transmission can be predicted.

## 6. Disrupted seasonality and ecosystem health

Ecological studies have identified potential problems associated with disruption of seasonality, owing to the desynchronization of key seasonal interactions among wild species (figure 1*b*). However, our existing understanding of the ecological implications of disrupted seasonality is mostly based upon studies of impacts upon single predator–prey relationships (such as the mismatch that can develop between the timing of seasonal coat colours and the annual duration of snow cover [81] and more recently also on plant–pollinator interactions [82–86]). Importantly, reproductive success in many species increases when reproductive timing and peak food availability are matched. Examples include egg hatching date in piscivorous seabirds, such as the Atlantic puffin (*Fratercula arctica*) [87], and in winter moths (*Operophtera brumata*) relative to tree budburst [88]; or calving date relative to vegetation growth in caribou (*Rangifer tarandus*) [89] or roe deer (*Capreolus capreolus*; [90]). An example that has been developed in some detail is the reducing match between the timing of breeding of forest songbirds, such as great tits (*Parus major*) and blue tits (*Parus caeruleus*), and the time that their prey, caterpillars feeding on oak leaves, are most abundant ([19,91–93]; electronic supplementary material, figure S5). Great tits that are most mismatched with the food peak have the fewest surviving offspring [94–96]. These ecological studies provide clear examples of how disrupted seasonality can affect the fitness of individuals living in an environment in which internal and external rhythms no longer match. The studies give numerous insights into how disrupted seasonality might also affect human and agricultural health.

Disrupted seasonality in natural systems can also be expected to affect ecosystem health. However, the effects at the ecosystem scale are more poorly understood than those at the individual level, because most studies have adopted the paradigm of the food chain (e.g. a single consumer population and a single resource species). While this has rendered the problem more tractable, patterns of species interaction in nature are in fact complex networks. Therefore, a major challenge is to move beyond relatively simple, mostly pairwise ecological interactions to consider the consequences of disrupted seasonality on population and community dynamics within broader, multispecies, interaction networks that include humans.

Despite the limitations of studies on dyadic interactions, there are already some clues that disrupted seasonality may affect ecosystem health. Under global change, the flowering phenology of plants and the seasonal activity phase of pollinators may shift to a different extent or even in opposing directions (electronic supplementary material, figure S2), thereby potentially leading to temporal mismatches and the disruption of existing interactions [97–99]. However, recent studies based on long-term phenology data indicate that bee emergence keeps pace with advanced plant-flowering, at least under current climatic conditions and for generalist plant–pollinator interactions [100]. Importantly, species-rich pollinator communities may also be able to buffer negative consequences of global warming [101], because plant pollinator networks exhibit plasticity with lost interactions being capable of replacement by new ones [102]. This underpins the importance of sustaining biodiversity for mitigating the impact of global climate change. Clearly, while there is ample scope for disrupted seasonality to strongly affect ecosystems, the few existing examples suggest that at present the sum impacts appear relatively small. In vertebrates, genetic variation in seasonality in wild populations of mammals and birds may not be sufficient to track changes in climate [103,104]. It remains to be seen if our collective perspective will hold once long-term research has been conducted as global climate change continues.

## 7. Disrupted seasonality and agricultural health

Ecological, physiological and epidemiological considerations suggest that seasonal disruption could also lead to substantial problems in agricultural industries, impacting both crop production and livestock viability, and hence, food security. The two most prevalent drivers of altered crop yields are changes in pollination and pest infestation. Notably, 70% of major crops and 35% of global crop production volume [82], with an estimated global economic value of $189 billion (€153 billion) per year [105], depend on seasonal pollination by bees and other insects. The spread of pest insects is a significant threat to human food security. For example, aphid outbreaks are expected to intensify owing to extended growing seasons (figure 1*c*) permitted by altered seasonal environmental conditions (reviewed by Bale & Hayward [106]). The peach–potato aphid and the grain aphid are examples of vectors of devastating plant virus diseases. In the past 20 years, increased occurrences of mild winters have resulted in earlier spring migrations of the winged form of these aphids into crops during their most vulnerable stages, resulting in epidemic outbreaks. The complex interactions between crops, pests and pathogens in the context of climate change urgently need more research [107].

Neglect of seasonal physiology and of the consequences of its disruption, negatively impact livestock health. Agricultural industries, notably the poultry industry, diminish seasonality by choosing light and temperature conditions to maximize reproduction and growth and thus profitability on an industrial scale (figure 1*d*). Some livestock, especially hens (*Gallus gallus*), may therefore be instructive models of longer-time effects of aseasonal conditions. Without the opportunity to seasonally pause laying and regenerate, notably by moulting [108], these hens become morbid and are typically slaughtered; only by allowing or forcing a moult can these effects be reversed. In other species used for human food production, breeding practices that select for animals which are capable of year round reproduction have also reduced seasonal rhythms. Farmed cattle (*Bos primigenius taurus*) have the ability to reproduce year round, but despite selection for decreased seasonal physiology, some ancient patterns remain, including the expression of poorly understood genes relating to hibernation and seasonal biology [109]. Many sheep breeds (*Ovis aries*) are excellent models for seasonal reproduction (electronic supplementary material, figure S6*a*) because they exhibit marked seasonal changes that are driven by internal rhythms synchronized by environmental factors [4]. Similar to the seasonal variation in humans discussed above, both sheep and cattle exhibit seasonal changes in disease diagnoses (electronic supplementary material, figure S6*b*).

To exemplify the possible implications of seasonal biology for morbidity and management of livestock, we highlight provisional data from passive surveillance in the UK on nutritional diseases (electronic supplementary material, figure S6*b*). Selenium (Se)-related deficiencies are common in farmed ruminants [110], so that sheep and cattle are commonly given supplementary Se. This perceived risk of deficiency and consequent timing of supplementation is driven by expected environmental supply and climatic- or management-driven challenge rather than by knowledge of underlying physiological processes that drive vulnerability. The contrasting peak months of diagnosis of Se deficiency syndromes in sheep versus cattle (electronic supplementary material, figure S6*b*) could be driven by different factors such as livestock management or disruptions in internal annual rhythms. Se deficiency is apparent in humans and is directly linked to viral pathology [111]. Seasonal variations in Se deficiency or supplementation in agriculture could, therefore, have significant implications for livestock health and human food systems.

## 8. Concluding remarks

Although our appreciation of the critical role of seasonal biology in the health and welfare of organisms (including humans), natural ecosystems and industries is growing, our ignorance of the effects of seasonal disruption on these systems is profound. We call for the development of an integrated, interdisciplinary, seasonality-focused research agenda (electronic supplementary material, table S3), inspired by a one health approach [24]. We propose three primary aims: understanding the internal seasonal clock, studying seasonality in human and veterinary clinical settings, and gaining ecological network perspectives.

### (a) Understanding the internal seasonal clock

The mechanistic basis of the seasonal clock, including its conserved and variable elements across vertebrates, remains largely unknown. This is in contrast to remarkable advances in our understanding of the circadian clock where the expanding knowledge of individual genetic variation is paving the way to personalized medicine [112]. Obtaining a similar level of detail about genes and physiological processes involved in the seasonal timing mechanisms and functional variation in these genes, would aid in identifying people, livestock and crops susceptible to the impact of seasonal disruption. This research should include genomic and transcriptomic investigation of seasonal biology across seasonal gradients in nature and also long-term studies of human and animal health. The current research focus on short-lived animal models offers limited answers for long-term health management of humans, relative to the value of incorporating studies of long-lived seasonal species. Beyond animal systems, manipulating plant clocks might also enable the development of crops (by either artificial selection or transgenic approaches) that are more resistant to temporal mismatches owing to climate change, preserving and possibly improving crop production systems.

### (b) Human and veterinary clinical settings

Seasonal patterns in health and physiology can be powerful indicators of possible underlying pathways, e.g. the neuroendocrine regulation of metabolism and obesity [113]. Data from human and veterinary pathology are often poorly integrated. Both are frequently only locally available, making it difficult to gain a broad understanding and to identify possible aetiologies. For example, revealing the seasonality of human diseases is now becoming possible as powerful epidemiological approaches are developed [33], but data often need to be tediously compiled from dispersed and poorly accessible sources. We encourage unified collection of seasonal health data, including possible use of big data analytics [114]. Analysis using improved empirical and epidemiological tools can help to identify drivers of seasonality (e.g. through trends in ambient temperature, latitude and day length) and also seasonal periods of particular vulnerability.

### (c) Gaining ecological network perspectives

Because organisms are sensitive to changes in the rhythms of species with which they interact, consequential mismatches propagate across ecological networks. Most research focuses on pairwise predator–prey, plant–herbivore or plant–pollinator interactions, but pairwise interactions need to be scaled up to more complex food webs and host–vector–pathogen systems. We emphasize the value of longer-term monitoring in ecological studies, with a view of multiple components and ‘neighbours’ in the network of species of particular interest. There is a real need for large-scale experimental approaches to understand the ecosystem-level consequences of shifted or disrupted seasonal timing.

Overall, in our view: (i) understanding the internal seasonal clock, (ii) enhancing seasonal analyses in human and veterinary settings, and (iii) integrating data across ecological and agricultural networks, will enable an integrative platform for addressing seasonal disruptions in a rapidly changing world.

## Supplementary Material

Figures S1-S6 and Tables S1-S3

## Data accessibility

The review does not contain new data; all work was obtained from previously published material.
