# Supplementary material for: Disrupted seasonal biology impacts health, food security and ecosystems
Source: Proc Biol Sci. 2015 Oct 22;282(1817):20151453. doi: 10.1098/rspb.2015.1453 (PMC4633868; doi:10.1098/rspb.2015.1453)
Supplement: Figures S1-S6 and Tables S1-S3 [file rspb20151453supp1.pdf]

Supporting Information: Disrupted seasonal biology impacts health, food security, and ecosystems

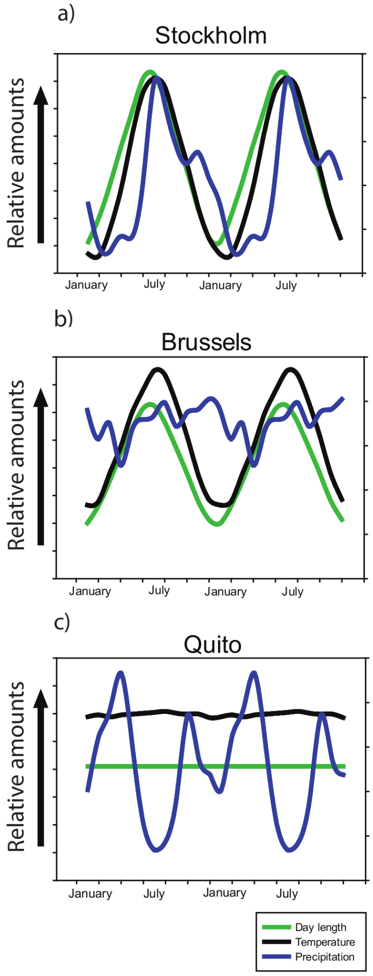

Fig. S1 - Naturally occurring seasonal cycles in day length, temperature and precipitation from three geographically distinct cities. a) Stockholm experiences marked changes in all three climate factors that are synchronized across the year providing an extremely predictable cycle. b) Brussels exhibits synchrony between day length and temperature, but the changes are less pronounced compared to Stockholm. c) Quito lacks substantial changes in day length and temperature, yet exhibits marked bimodal periods of seasonal precipitation. Climate change predictions indicate increased temperatures over the next several decades, but the impeding variation will not affect the world in a uniform manner and will instead have localized effects on flora and fauna.

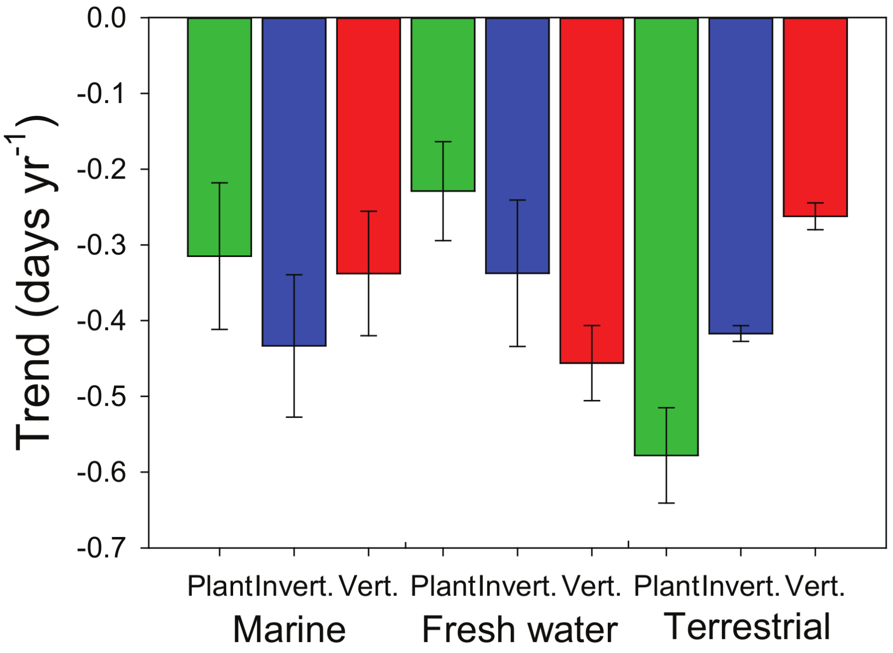

Fig. S2 - Changes in phenology of plants, invertebrates and vertebrates. Data are Mean +/- SEM rates of changes for marine, fresh water and terrestrial environments in the UK during the periods encompassing 1976–2005. All organisms and environments show negative trends, suggesting an advance in seasonal events. Data are taken from Thackeray et al., 2010, (26) from main text.

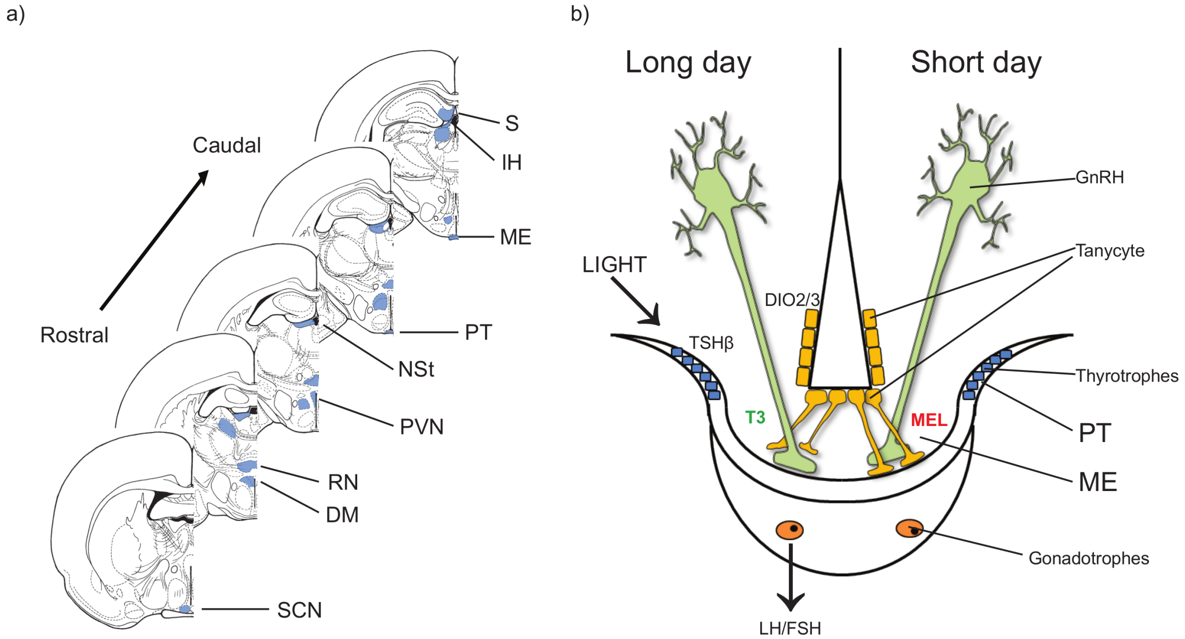

Fig. S3 - Light dependent regulation of seasonal rhythms. The nocturnal secretion of the pineal gland hormone melatonin (MEL) codes night length in all known vertebrate species. Annual changes in day length lead to short (long days in summer) or long (short days in winter) durations in MEL secretion. MEL acts both centrally and peripheral by binding MEL receptors. a) MEL receptor is widely distributed in the brain, this schematic provides the general distribution using a rodent brain. Cross sections of the rodent brain with MEL receptor regions indicated in blue. Abbreviations: dorsomedial nucleus (DM), lateral habenula (IH), nucleus of the stria terminalis (NSt), paraventricular nucleus (PVN), reunions nucleus (RN), subiculum (S), suprachiasmatic nucleus (SCN). b) The pars tuberalis (PT) and median eminence (ME) in the ventral hypothalamus are the key brain regions for the seasonal regulation of physiology and behaviour in many species. Long day lengths ($>$ 12 hrs, left side of figure) stimulate thyrotrophes in the PT to produce thyrotrophin-stimulating hormone β (TSHβ). Increased TSHβ alters the ratio of thyroid hormone enzymes (deiodinase Type II and III, DIO2 and DIO3) leading to the increased local synthesis in triiodothyronine (T3). T3 then permits the release of gonadotropin-releasing hormone1 (GnRH) which in turn stimulates gonadotrophes to release luteinizing hormone (LH) and follicle-stimulating hormone (FSH). LH and FSH induce gametogenesis and steroidogenesis facilitating reproductive physiology and behaviour. In short days—right side of figure—increased MEL duration inhibits TSHβ expression, reversing the ratio of DIO2/3, reducing local T3 concentrations and inducing gonadal involution.

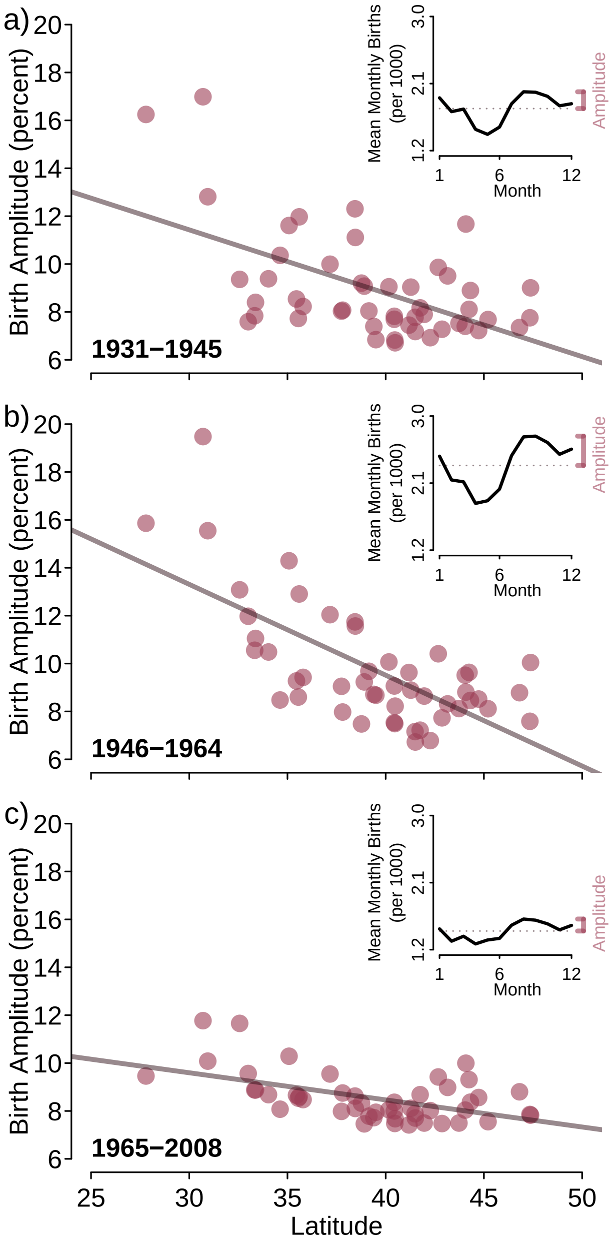

Fig. S4 - Seasonal birth pulses in the 20th century USA. Each year there are seasonal fluctuations in the birth rate. As an example, the insets show the seasonal fluctuations in birth rate for the state of Louisiana ($31°$N). For each state, the size of the seasonal birth pulse was measured as the amplitude (the percent deviation from the mean birth rate). The amplitude is a measure of the difference between maximum births and mean births. The main panels show that the birth amplitude changes with latitude: southern states have significantly larger birth amplitude than northern states. Furthermore, the amplitude was higher during the pre-baby boom (panel a) and baby boom (panel b) eras, but diminished in the contemporary era (panel c). Figure reproduced from (33) in the main text.

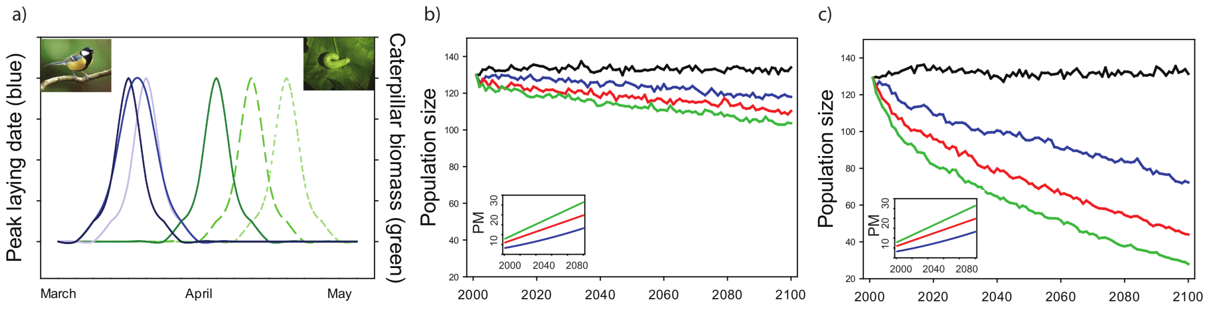

Fig. S5 - Mismatches in annual rhythms from an ecological perspective. Annual rhythm in great tit (*Parus major*) reproduction is timed to coincide the feeding of its nestlings with the peak in caterpillar biomass during the spring season. a) Environmental changes in external cues (i.e., temperature) have resulted in peak caterpillar biomass shifting earlier in the year, indicated by the leftward direction in the green distribution. However, great tit laying dates, indicated by the blue distribution, have not shifted peak values at a similar rate, resulting in a significant mismatch and consequently a seasonal disruption that affects the number and weight of great tit fledglings. b) In silico models to investigate stochastic population dynamics under various scenarios of mismatched rhythms (black: no population mismatch; blue: mild; red: moderate; green: strong) have revealed slight decreases in great tit populations with the strongest decline under the most extreme scenarios. The lack of severe decreases in population numbers results from a reduction in intraspecific resource competition. c) When simulations are conducted assuming density-independence, the impact of seasonal mismatch in rhythms is significantly enhanced leading to potentially marked decreases in wild populations. Inset graphs located in panels (b) and (c) show trends in population mismatch associated with the respective mismatch scenarios. Data are adapted from (22) and (92) in main text.

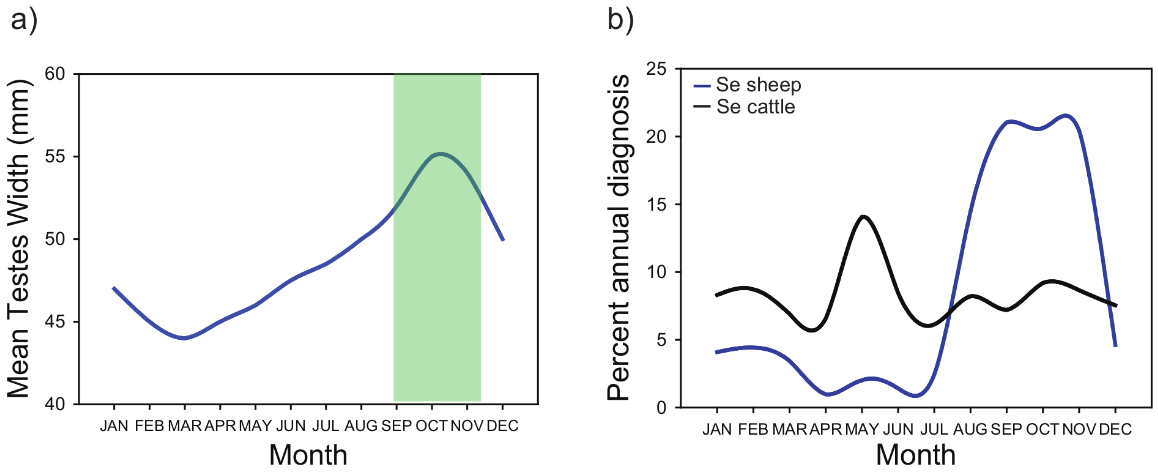

Fig. S6 - Seasonal variations in animal reproductive state and health. a) Domestic sheep are one of the main model species for the neuroendocrine mechanisms governing seasonal rhythms. Sheep are short-day breeders and breed during the fall (indicated in green bar), so that lambing is timed to coincide with spring pasture growth in high latitudes. b) The percentage of diagnoses in selenium deficiency (Se) in sheep (blue) and cattle (black) exhibit marked seasonal patterns. Data are adapted from (a) Lincoln GA, Short RV (1980) Rec Prog Horm Res 36:1-52 and (b) extracted from VIDA reports of Defra Animal Health and Veterinary Laboratory Agency (AHVLA), (`http://www.defra.gov.uk/ahvla-en/category/publications/disease-surv/vida/`)

Table S1 - Seasonal peaks in illness and disease in humans by month of birth. Data adapted from (17) in main text. Red and blue color denotes increased prevalence in northern and southern latitudes, respectively.

Condition	Month of Birth											
	Jan	Feb	Mar	Apr	May	Jun	Jul	Aug	Sept	Oct	Nov	Dec
General Pathologies												
Crohn's Disease (Israel)												
Hodgkins Disease												
Asthma (Denmark)												
Childhood Diabetes mellitus												
Glaucoma												
Asthma (UK)												
Psychiatric Disorders												
Schizoaffective Disorder												
Bipolar												
Neuroses												
Eating Disorder												
SAD												
Personality Disorder												
Alcohol Abuse												
Autism												
Schizophrenia (N. Hemisphere)												
Schizophrenia (S. Hemisphere)												
Neurological Illnesses												
Epilepsy												
Alzheimer's Disease												
Narcolepsy												
Amyotrophic Lateral Sclerosis												
Mental Retardation												
Parkinson's Disease												
Motor Neuron Disease												
Down's Syndrome												
MS (Northern Hemisphere)												
MS (Southern Hemisphere)												

Table S2 - Seasonal variation in immune activity

Species	Immune assay	Arm of Immune System	During Winter (or short days)	Reference
**Mammals**				
Spermophilus beldingi	SRBC antibodies	humoral	decrease	Sidky *et al*. (1972)
Clethrionomys glareolus	SRBC antibodies	humoral	increase	Saino *et al*. (2000)
Sigmodon hispidus	spleen PFC to SRBC	cell mediated	increase	Lochmiller *et al*. (1994)
Peromyscus maniculatus	SNV antibodies	humoral	decrease	Lehmer *et al*. (2010)
	splenocyte proliferation	cell mediated	increase	Demas & Nelson (1998)
Funambulus pennanti	Concavalin A and DTH	cell mediated	increase	Ahmad & Haldar, 2009
Mesocricetus auratus	splenocyte proliferation	cell mediated	increase	Brainard *et al*. (1987)
Rattus norvegicus	CD8+ and CD3+ lymphocytes	adaptive	increase	Prendergast et al, 2007
Phodopus sungorus	lymphocyte proliferation	cell mediated	decrease	Yellon *et al*. (1999)
	sickness responses	integrative	decrease	Baillie & Prendergast, 2008
	wound healing	integrative	decrease	Kinsey et al, 2003
	leukocyte ROS production in response to zymosan	cell mediated	decrease	Pawlak et al, 2009
	DTH to DNFB	cell mediated	increase	Bilbo *et al*. (2002)
	anti-KLH IgG	humoral	decrease	Zysling et al, 2009
	NK cell cytolytic activity	innate	increase	Yellon *et al*. (1999)
	phagocytosis	innate	decrease	Yellon *et al*. (1999)
	fever	innate	decrease	Fonken et al, 2012
	fever	innate	decrease	Ashley et al, 2012
**birds**				
Perdicula asiatica	total lymphocyte count	adaptive	increase	Kharwar & Haldar (2011)
Gallus gallus	peritoneal leukocytes	cell mediated	decrease	Turkowska et al. (2013)
Sturnus vulgaris	splenocyte proliferation	cell mediated	increase	Bentley *et al*. (1998)
Philomachus pugnax	DTH to PHA	cell mediated	increase	Lozano & Lank (2003)
Passer domesticus	wound healing	integrative	decrease	Kinsey *et al*. (2003)
	DTH to PHA	cell mediated	increase	Greenman *et al*. (2005)
	DTH to PHA	cell mediated	no change	Gonzalez *et al*. (1999)
	DTH to PHA	cell mediated	increase	Martin *et al*. (2004, 2005, 2006*a*)
Parus major	heterophil/lymphocyte ratio	cell mediated	decrease	Pap et al, 2010
Zonotrichia leucophrys	fever	innate	decrease	Owen-Ashley & Wingfield (2006)

Table S3 - Questions and Opportunities from a One-health perspective.

**1) Mechanisms underlying health.** The neural mechanisms that integrate the disparate environmental cues or generate intrinsic rhythmicity are not well described. Uncovering the mechanisms that underlie the generation and regulation of seasonal physiology and immune function is of fundamental importance. Advances in next generation technologies are an important step to identify molecular and cellular pathways involved in internal control of seasonal rhythms. This will also help assess the relative flexibility of species and their predicted response to seasonal disruption.
**2) Human health.** Despite efforts to artificially maintain a constant, "eternal summer" environment, humans still exhibit seasonal rhythms in physiology, immunity and behaviour. Acknowledgement of these patterns is essential for developing an informed approach to treating seasonally recurring illnesses. Furthermore, local changes in environmental seasonality due to climate change will require the development of medical practices that focus on impacts to local populations.
**3) Ecosystem health.** Climate change models predict a mean increase in global temperatures that will have localized impacts. Further insight into the mechanisms and functional consequences of mismatch within ecological networks will be essential for predicting population dynamics and selection pressures for animal fitness and conservation efforts.
**4) Agriculture and Food-security.** Food security is rapidly becoming a national and international concern. Seasonal variation in crop and livestock productivity has direct influence on human health and welfare. Developing a comprehensive approach that emphasizes mechanistic knowledge and industrial practice will be necessary to reduce risks associated with animal morbidity and mortality due to seasonal disease prevalence.
